# Assessment of Risk Factors for Surgical Site Infection in Diabetic Patients Undergoing Elective Surgery

**DOI:** 10.7759/cureus.108920

**Published:** 2026-05-15

**Authors:** Muhammad Sameer Anwar, Rimsha Malik, Hafiz Muhammad Shoaib, Javed Ahmed, Sumaira Hassan, Muhammad Atta Ullah, Daphnie Fernando, Abdul Ahad Mehboob, Zahra Ali

**Affiliations:** 1 Medicine, Hayat Memorial Teaching Hospital, Lahore, PAK; 2 Department of Medicine, Allama Iqbal Medical College, Lahore, Lahore, PAK; 3 Pediatric Surgery, Children Hospital Lahore, Lahore, PAK; 4 Surgery, Avicenna Medical College and Hospital Lahore, Lahore, PAK; 5 Medical School, Pakistan Naval Station (PNS) Shifa Hospital, Karachi, PAK; 6 General Surgery, Army Hospital Colombo 5, Colombo, LKA; 7 Department of Pathology, National Laboratory and Testing Centre, Lahore, Lahore, PAK; 8 Obstetrics and Gynaecology, Sir Ganga Ram Hospital, Lahore, Lahore, PAK

**Keywords:** diabetes mellitus, elective surgery, glycemic control, surgical site infection, wound classification

## Abstract

Background: Surgical site infections (SSIs) remain a major postoperative complication, particularly among diabetic patients who are inherently at higher risk due to impaired immunity and delayed wound healing.

Objective: To assess the risk factors associated with SSIs in diabetic patients undergoing elective surgical procedures.

Methods: This retrospective study was conducted at Children Hospital Lahore, from April 2024 to April 2025. A total of 150 diabetic patients scheduled for elective surgery were enrolled using non-probability consecutive sampling. Preoperative, intraoperative, and postoperative variables were recorded, including glycemic indicators, comorbidities, operative duration, wound classification, antibiotic timing, and postoperative wound status.

Results: The mean age of participants was 57.8 ± 11.4 years, and 58.7% were male. The overall SSI rate was 25.3% (n = 38). Higher HbA1c levels were significantly associated with SSI (8.4 ± 1.3 vs. 7.1 ± 1.0, p < 0.001). Operative duration of two or more hours (p = 0.02), contaminated wound classification (p = 0.01), smoking (p = 0.03), and incorrect antibiotic prophylaxis timing (p = 0.04) were also significantly correlated with infection. Logistic regression identified poor glycemic control (adjusted odds ratio (AOR) 3.1, 95% CI: 1.6-5.9), prolonged surgery (AOR 2.4, 95% CI: 1.1-4.8), contaminated wound class (AOR 2.9, 95% CI: 1.3-6.5), and smoking (AOR 1.9, 95% CI: 1.01-3.8) as independent predictors of SSI. Patients who developed SSI had significantly longer hospital stays (9.8 ± 3.1 vs. 5.3 ± 1.9 days, p < 0.001).

Conclusion: It is concluded that SSIs in diabetic patients are strongly linked to modifiable perioperative factors. Poor glycemic control, prolonged operative duration, contaminated wounds, and smoking significantly increase infection risk.

## Introduction

Surgical site infections (SSIs) represent an unfinished complication in contemporary surgical practice, being a leading postoperative issue and a global health issue as well [1]. They account for over 20% of all healthcare-acquired infections and are the primary cause of many unanticipated post-surgical admissions. Their consequences consist of prolonged hospital stays, complications that could become deadly, exacerbated financial expenses, and extended recovery times [2]. The challenges posed by SSIs are not solely medical. SSIs also create economic and psychological ramifications. Diabetes significantly changes the physiology of wound healing by creating a hyperglycemic state, which slows the immune processes of chemotaxis, phagocytosis, and oxidative bursts (i.e., the immune system itself becomes sluggish) [3].

Microangiopathy reduces the flow of oxygen and nutrients to the tissues, creating a perfect environment for bacterial growth and a hostile environment for tissue repair. The delay of collagen synthesis and reduction of fibroblast activity slow down the overall healing of the wound [4]. This, combined with the increased presence of obesity, renal dysfunction, neuropathy, and peripheral vascular disease in diabetic individuals, creates surgical wounds that are prone to becoming infectious [5]. These are the reasons for the up to two-to-three-fold increased rates of SSIs in diabetic individuals, even under optimum conditions for surgery, as when one would do an elective surgery. The SSI literature suggests these types of infections can develop during procedures 3% to 20% of the time, and possibly more with high-risk patients. SSIs also represent growing tissue damage and negative wound healing outcomes that lead to more morbidity and lifelong disabilities [6]. This growing SSI morbidity also drives and complicates the overall healthcare costs. There are also some misconceptions among surgeons in that SSIs are not a trivial and benign condition [7].

The Centers for Disease Control and Prevention (CDC) International Classification of Diseases (ICD) describes an SSI as an infection that occurs in the area of a surgical wound in the 30 days that follow the procedure and includes some sign of infection, including swelling and pus [8]. The CDC has defined four classes of surgical procedures, with class I having the lowest risk and class IV having the highest. Electively scheduled procedures are class I [8,9]. It is during these procedures that the clinician has complete control to optimize the patient’s comorbidities and follow a standardized procedure [10]. Unfortunately, this also describes many diabetic patients undergoing elective procedures with poor glycemic control, significant comorbidities, and little education on postoperative care [11]. The preoperative period, which includes these patients, is also one of the most important yet most often overlooked periods of time for a planned intervention. Postoperative outcomes can be positively influenced by engaging in some practices, for instance, early screening, refining blood sugar levels, stopping smoking, determining nutritional status, managing weight, and evaluating patients for peripheral vascular disease [12]. The same can be said for some intraoperative practices that can be essential to patients being protected from infections. These include, but aren’t limited to, thorough antimicrobial prophylaxis, preservation of normothermia, effective surgical method, and shorter time in the procedure [13].

Our objective was to assess the risk factors associated with SSIs in diabetic patients undergoing elective surgical procedures.

## Materials and methods

This retrospective study was conducted at Children Hospital Lahore (a tertiary care teaching hospital), from April 2024 to April 2025. A total of 150 diabetic patients were included using a non-probability consecutive sampling technique. Adult patients aged 18 years or older with a confirmed diagnosis of type 1 or type 2 diabetes mellitus who underwent elective clean, clean-contaminated, or contaminated surgical procedures and had complete preoperative, intraoperative, and postoperative records available were included in the study. Emergency surgical cases, patients with any pre-existing infection, immunocompromised individuals such as those receiving long-term corticosteroid therapy or chemotherapy, and patients with incomplete medical records were excluded.

Data collection procedure

Data were collected using a structured proforma designed specifically for this study. Research team members extracted data from patient databases such as medical records, operative notes, anesthesia records, and postoperative assessments. Each entry was validated and checked by the principal investigator before the data were processed for analysis. During the preoperative assessments, the staff recorded data on patients' demographic profiles such as age, sex, and body mass index. The staff also collected average diabetes duration, other diabetes-related data like fasting and random blood glucose and HbA1c levels, and diabetes symptoms, if any. Other health issues, such as hypertension, ischemic heart disease, chronic kidney disease, and peripheral vascular disease, along with smoking and nutritional status, were recorded as well. Staff also collected data on the timing of prophylactic antibiotics and the method of surgical skin prep, as these are also known to affect the risk of infection. Data collected intraoperatively included the procedure performed, American Society of Anesthesiologists (ASA) physical status score, and the total surgical time, which was categorized as less than two hours or two or more hours. These scores are relevant to SSIs in diabetic patients because they reflect glycemic control, overall patient health status, and operative risk factors that directly influence immune function, wound healing, and susceptibility to postoperative infection. Other recorded variables include wound class, temperature regulation during the procedure, and continuous glucose monitoring. The degree to which surgical crew members followed aseptic practices was also assessed, since these factors are known to affect the infection risk. The primary focus of early postoperative monitoring was on glycemic control within the first two days after surgery. Other factors being evaluated included the methods for applying and changing the dressings. Information was also recorded on the length of the hospital stay and any complications that may have arisen after surgery. Using the guidelines established by the CDC, the presence of SSIs was determined and categorized as either superficial, deep, or organ/space infection [8]. Daily assessments of the patients for infection included the presence of erythema, purulent discharge, fever, tenderness, or pain, and slow healing of the affected area.

Data analysis

Data analysis was conducted using SPSS v26.0 (IBM Corp., Armonk, NY, USA). Descriptive statistics were computed for baseline characteristics. The chi-square test was applied to categorical variables to assess associations with SSI development, while continuous variables were analyzed using the independent sample t-test. Multivariate logistic regression analysis was performed to identify independent predictors of SSIs. Statistical significance was set at p < 0.05.

## Results

Data were collected from 150 patients. The mean age of the participants was 57.8 ± 11.4 years, with a male predominance of 58.7%. The average BMI was 28.6 ± 4.9 kg/m², indicating that most patients were overweight. The majority of participants had type 2 diabetes (90%), while only 10% had type 1 diabetes, and the median duration of diabetes was nine years (IQR: 5-14). Poor long-term glycemic control was common, with 62.7% of patients having HbA1c levels above 7%. A total of 21.3% were active smokers. Hypertension was the most prevalent comorbidity (61.3%), followed by ischemic heart disease (25.3%), chronic kidney disease (14.7%), and peripheral vascular disease (12.7%). The mean duration of surgery was 118 ± 39 minutes, and 38.7% of patients had operative times equal to or exceeding two hours. Higher surgical risk was observed in 42.7% of participants with ASA scores of III or above. Adherence to perioperative protocols was generally strong, with 82.7% receiving prophylactic antibiotics within the recommended 60-minute window, 92% maintaining normothermia intraoperatively, and 96% undergoing intraoperative glucose monitoring (Table 1).

**Table 1:** Baseline Characteristics of Diabetic Patients Undergoing Elective Surgery (n = 150) ASA: American Society of Anesthesiologists

Variable	Value
Age (years), mean ± SD	57.8 ± 11.4
Gender	
Male	88 (58.7%)
Female	62 (41.3%)
BMI (kg/m²), mean ± SD	28.6 ± 4.9
Type of Diabetes	
Type 1	15 (10.0%)
Type 2	135 (90.0%)
Duration of Diabetes (years), median (IQR)	9 (5–14)
HbA1c >7%	94 (62.7%)
Smoking	32 (21.3%)
Comorbidities	
Hypertension	92 (61.3%)
Ischemic heart disease	38 (25.3%)
Chronic kidney disease	22 (14.7%)
Peripheral vascular disease	19 (12.7%)
Wound Classification	
- Clean	72 (48.0%)
- Clean-contaminated	54 (36.0%)
- Contaminated	24 (16.0%)
Operative Duration (minutes), mean ± SD	118 ± 39
Operative Duration ≥2 hours	58 (38.7%)
ASA Score ≥ III	64 (42.7%)
Antibiotic Prophylaxis Within 60 Minutes	124 (82.7%)
Temperature Maintenance Intraoperatively	138 (92.0%)
Intraoperative Glucose Monitoring	144 (96.0%)

The overall SSI rate was 25.3% (n = 38). Among these infections, superficial SSIs were the most common (57.9%), followed by deep incisional infections (28.9%) and organ/space infections (13.2%) (Table 2).

**Table 2:** Frequency and Type of Surgical Site Infections (SSIs) (n = 150)

Variable	Value
Overall SSI Rate	38 (25.3%)
SSI Classification	
• Superficial	22 (57.9%)
• Deep incisional	11 (28.9%)
• Organ/space	5 (13.2%)

Individuals who developed SSI had significantly higher mean HbA1c levels (8.4 ± 1.3 vs. 7.1 ± 1.0; p < 0.001), indicating that poor glycemic control was strongly associated with postoperative infection. Prolonged operative duration (two or more hours) was also significantly more frequent in the SSI group (60.5% vs. 31.3%; p = 0.02). Contaminated wound class was identified more often among patients with SSI (31.6% vs. 10.7%; p = 0.01). Smoking was another significant risk factor, seen in 34.2% of the infected patients compared to 17.0% of those without SSI (p = 0.03). Incorrect timing of antibiotic prophylaxis also contributed significantly to SSI occurrence (31.6% vs. 12.5%; p = 0.04) (Table 3).

**Table 3:** Comparison of Risk Factors Between Surgical Site Infection (SSI) and Non-SSI Groups

Risk Factor	SSI Present (n = 38)	SSI Absent (n = 112)	Test Statistic	p-value
HbA1c (mmol/mol) (mean ± SD)	8.4 ± 1.3	7.1 ± 1.0	t = 5.52	<0.001
Operative Duration ≥2 hours	23 (60.5%)	35 (31.3%)	χ² = 7.77	0.02
Contaminated Wound Class	12 (31.6%)	12 (10.7%)	χ² = 7.83	0.01
Smoking	13 (34.2%)	19 (17.0%)	χ² = 4.70	0.03
Incorrect Antibiotic Timing	12 (31.6%)	14 (12.5%)	χ² = 6.08	0.04

Poor glycemic control (HbA1c >7%) remained a strong independent risk factor (adjusted odds ratio (AOR) 3.1; 95% CI 1.6-5.9; p = 0.001). Operative duration of two or more hours was also independently associated with a higher risk of SSI (AOR 2.4; 95% CI 1.1-4.8; p = 0.02). Similarly, contaminated wound class (AOR 2.9; 95% CI 1.3-6.5; p = 0.01) and smoking (AOR 1.9; 95% CI 1.01-3.8; p = 0.04) remained significant predictors after adjustment (Table 4).

**Table 4:** Multivariate Logistic Regression for Independent Predictors of Surgical Site Infection (SSI)

Variable	Adjusted Odds Ratio (AOR)	95% CI	p-value
Poor glycemic control (HbA1c >7%)	3.1	1.6–5.9	0.001
Operative duration ≥2 hours	2.4	1.1–4.8	0.02
Contaminated wound class	2.9	1.3–6.5	0.01
Smoking	1.9	1.01–3.8	0.04

Fever was significantly more common in the SSI group (55.3% vs. 15.2%; p < 0.001), as was postoperative hyperglycemia exceeding 200 mg/dL within the first 48 hours (71.1% vs. 34.8%; p < 0.001). Patients with SSI were more likely to require reoperation (10.5% vs. 1.8%; p = 0.03) and IV antibiotic therapy (81.6% vs. 21.4%; p < 0.001). Additionally, SSI patients had a substantially longer hospital stay and a higher 30-day readmission rate (15.8% vs. 2.7%; p = 0.01), indicating the significant clinical impact of postoperative infections (Table 5).

**Table 5:** Postoperative Outcomes in Patients With and Without Surgical Site Infection (SSI) Chi-square test was applied for categorical postoperative outcomes (fever, hyperglycemia, reoperation, IV antibiotic requirement, 30-day readmission). An independent samples t-test was applied for the continuous variable (hospital stay in days). A p-value ≤0.05 was considered statistically significant.

Outcome	SSI Present (n = 38)	SSI Absent (n = 112)	Test Statistic	p-value
Fever	21 (55.3%)	17 (15.2%)	χ² = 21.98	<0.001
Hyperglycemia >200 mg/dL (first 48 hrs)	27 (71.1%)	39 (34.8%)	χ² = 14.67	<0.001
Need for reoperation	4 (10.5%)	2 (1.8%)	χ² = 4.62	0.03
Need for IV antibiotics post-op	31 (81.6%)	24 (21.4%)	χ² = 46.82	<0.001
Hospital stay (days), mean ± SD	9.8 ± 3.1	5.3 ± 1.9	t = 9.02	<0.001
30-day readmission	6 (15.8%)	3 (2.7%)	χ² = 7.64	0.01

## Discussion

This research aimed to identify the main predictors of surgical site infections among diabetics undergoing elective surgeries. The study was able to establish that inadequate glycemic control, extended surgical time, higher wound contamination class, and smoking were key predictors. The 25.3% infection rate among participants was considerably above the average infection rates of postoperative SSIs among non-diabetic patients. The 25.3% SSI rate observed in this study is higher than rates reported among non-diabetic patients due to diabetes-related factors such as impaired immunity, poor wound healing, and perioperative hyperglycemia. Additionally, single-center studies often report higher infection rates compared with global data because they may include more complex or high-risk cases and reflect local variations in patient profiles and healthcare resources. This shows that diabetes increases the infection risk even with the best perioperative care. This is in support of the data from other regions of the world, which shows diabetes to be a risk factor for SSIs as they have higher rates (2 to 3 times) than non-diabetic patients.

This is secondary to ineffective host defense mechanisms, as well as slower wound healing. The most salient risk factor for infection in this study was certainly poor glycemic control, higher HbA1c levels, and infection. This consistency in hyperglycemia and immune compromise is no surprise, as higher glucose levels are known to inhibit neutrophil chemotactic, phagocytic, and oxidative burst responses as well as to reduce collagen availability and delay re-epithelialization [14]. The adjusted odds ratio for HbA1c >7% is similar to that seen in multiple studies around the world on the importance of metabolic optimization. The fact that plenty of patients went ahead and had elective surgery even with poor glycemic control points to an important clinical oversight: the preoperative phase is not properly used for risk mitigation. This is the type of controllable factor that should have been caught by today’s perioperative systems. A significant association with SSI was also seen with operative time of two hours or more [15]. The longer the surgery, the greater the risk of exposure to additional bacterial contamination, tissue trauma, fluid shifts, and compromised wound integrity. This finding is in line with studies that show a doubling of the risk of SSI for cases that have a longer operative time than the scheduled benchmarks for a procedure [16].

Under what circumstances the procedure took a long time in this cohort is not known, so the operative time does not reflect a wide range of surgical complexity, or prolonged time for anesthesia, or even delay from the operating room, but it does emphasize the need for more effective intraoperative collaboration between surgical and anesthesia teams. The higher microbial load attributable to contaminated wounds cannot spell anything other than a strong risk factor for SSI, and yet the sharp rise in SSI rates associated with these cases relative to the clean-contaminated ones is particularly striking [17]. This makes all the more significant the need for antibiotic stewardship, stringent aseptic technique, and postoperative wound surveillance in the more contaminated wound classes. While some bacterial exposure is inevitable in all operative procedures, and particularly in the ones that are elective, the considerable rate of infection points to the need for a more individualized use of perioperative protocols on patients with diabetes complications in infected wounds. Smoking also remained independent of other risk factors in predicting the presence of SSI [18]. This is consistent with other work demonstrating that tobacco use is associated with reduced micro-vascular circulation, diminished oxygen delivery, and impaired immune response. The higher infection rate in patients with diabetes and smoking should motivate the adoption of a more rigorous approach to the documented smoking cessation strategies in the preoperative period, rather than mere recommendations, to structured cessation programs [19].

The multitude of risks interplaying with the diabetes diagnosed in these patients makes a strong case that elective surgery is anything but routine in this cohort. Instead, it requires a unique, individualized, risk-stratified approach bringing together metabolic optimization, careful surgical technique, and timely and vigilant postoperative follow-through. Notwithstanding improvement in surgical skills, SSIs are still far too prevalent even in elective cases. This indicates that a significant number of surgical services are still working with “standard protocols,” and are failing to adjust their perioperative approach to one that is patient-centered and individualized. This study should have a significant impact on how surgical services are organized. Strengthened preoperative screenings to optimize glycemic control, improvement and optimization of antibiotic timing, smoking cessation, and decreasing time in surgery could have a significant impact on reducing SSI rates. Furthermore, the introduction of multidisciplinary perioperative programs incorporating endocrinologists, surgeons, anesthetists, and nursing staff could address several of these risk factors rather than addressing them in a fragmented way. This study had several important limitations that should be acknowledged. First, its cross-sectional design restricted the ability to establish causal relationships between risk factors and the development of SSIs, limiting interpretation to associations only. Second, the use of non-probability sampling may have introduced selection bias, as the study population may not fully represent all diabetic surgical patients. Third, the data relied heavily on the accuracy and completeness of medical records, creating the potential for information bias, particularly regarding antibiotic timing, glycemic measurements, and perioperative care details.

## Conclusions

It is concluded that SSIs remain a significant postoperative challenge in diabetic patients undergoing elective surgery, with several modifiable factors contributing to increased risk. The study findings highlight that poor glycemic control, prolonged operative duration, contaminated wound classification, and smoking substantially elevate the likelihood of developing SSI. These results emphasize the need for strict perioperative metabolic optimization, improved adherence to antibiotic prophylaxis guidelines, and reinforcement of intraoperative efficiency and aseptic technique.
